# Intersectionality and Youth Identity Development Research in Europe

**DOI:** 10.3389/fpsyg.2020.00078

**Published:** 2020-01-31

**Authors:** Ursula Moffitt, Linda P. Juang, Moin Syed

**Affiliations:** ^1^Development of Identities in Cultural Environments, Department of Psychology, Northwestern University, Evanston, IL, United States; ^2^Diversity in Education and Development, Department of Inclusive Education, University of Potsdam, Potsdam, Germany; ^3^Narrative-Identity-Culture-Education, Department of Psychology, University of Minnesota, Minneapolis, MN, United States

**Keywords:** intersectionality, identity development, migration, ethnic-racial identity, youth identity, Europe, Islamophobia

## Abstract

The increasing application of intersectionality to the psychological study of identity development raises questions regarding how we as researchers construct and operationalize social identity categories, as well as how we best capture and address systems of oppression and privilege within our work. In the continental European context, the use of the intersectionality paradigm raises additional issues, since “race” was officially removed from the vernacular following the atrocities of WWII, yet racialized oppression continues to occur at every level of society. Within psychological research, participants are often divided into those with and without “migration background,” which can reiterate inequitable norms of national belonging while washing over salient lived experiences in relation to generation status, citizenship, religion, gender, and the intersection between these and other social locations. Although discrimination is increasingly examined in identity development research, rarely are the history and impact of colonialism and related socio-historical elements acknowledged. In the current paper, we aim to address these issues by reviewing previous research and discussing theoretical and practical possibilities for the future. In doing so, we delve into the problems of trading in one static social identity category (e.g., “race”) for another (e.g., “migration background/migrant”) without examining the power structures inherent in the creation of these top-down categories, or the lived experiences of those navigating what it means to be marked as a racialized Other. Focusing primarily on contextualized ethno-cultural identity development, we discuss relevant examples from the continental European context, highlighting research gaps, points for improvement, and best practices.

Across continental Europe, all mention of “race” was removed from official use following WWII, freezing the collective understanding of this social construct in the pseudo-scientific terms of the colonialist and Fascist eras (Goldberg, 2006; Möschel, 2011). For this reason, aside from the United Kingdom (U.K.) and Ireland, no European countries gather race-based population statistics, despite increasing diversity across the continent (Simon, 2012, 2017). The legal rationale for the removal of “race” is situated in colorblind ideology focusing on shared humanity, in direct response to the racial hierarchies created and used to justify the mass atrocities of the colonial and Fascist eras, including the Holocaust (Möschel, 2011). This erasure has hindered development of the collective understanding toward recognizing race as a social construct with ongoing material impact. That said, notions of who is European and who is Other (Bail, 2008; Essed and Trienekens, 2008; El-Tayeb, 2014) are naturalized through the use of national monikers (e.g., German, Belgian, Swedish) in opposition to “migrants,” often regardless of generation, citizenship, or self-identification. Despite race not being named, who is cast into which group is highly racialized, while also contingent upon religion, class, and additional social locations (Garner, 2007; Meer and Modood, 2009; Ramm, 2010; Korteweg, 2013; Bonjour and Duyvendak, 2018).

Thus, although “race” was officially removed from the vernacular, racism remains deeply embedded within continental European society, intersecting with anti-Muslim and anti-immigrant sentiment to inform norms, policies, and everyday interactions. In recent years, European sociologists and ethnic and women’s studies scholars (Lewis, 2013; Salem and Thompson, 2016; Boulila and Carri, 2017) have increasingly drawn on the paradigm of intersectionality to examine this phenomenon of racism without race. The majority of this work focuses on the structural level, offering critical analyses of intersecting systems of inequity, generally without empirically examining individual identity construction. For this reason, numerous scholars from across disciplines have called for a greater investigation into subjective perceptions of intersectional oppression and/or privilege (Essed, 1991; Yuval-Davis, 2006; Hulko, 2009). Yet, the study of identity development in continental Europe from an intersectional psychological perspective is not yet established.

In the U.S., on the other hand, an increasing number of psychologists have begun conducting individual focused research through an intersectional lens. Despite this trend, there is not yet consensus regarding what it can or should look like to apply intersectionality to psychological research. There is trepidation, which we share, regarding the most suitable application of a critical, social justice oriented, social constructionist paradigm in a field dominated by (post) positivist, quantitative research (Syed, 2010; Rosenthal, 2016; Moradi and Grzanka, 2017). As was discussed in the recent special issue of *New Directions for Child and Adolescent Development*, intersectionality is not a falsifiable theory of the kind generally applied within developmental science (Syed and Ajayi, 2018). Instead, it is a humanistic theory recognizing, questioning, and pushing against interlocking structures of inequity, including how they inform what is accepted as valid knowledge and what is understood as normative. So, echoing the question guiding the afore-mentioned special issue (Santos and Toomey, 2018), how can this paradigm be most appropriately applied to identity development research? In line with our focus, how can it be applied in continental Europe, where race is officially unacknowledged, though racialized inequity is a primary system of oppression shaping lived experience?

We argue that intersectional research can help shed light on precisely this issue, namely by examining how individuals are making sense of and pushing against the boundaries of ostensibly neutral and static social identity categories created in the past decades in the absence of “race.” As Cole (2009) laid out in her essay from which the questions guiding the *current* special issue were drawn, properly conducting intersectional research, including in psychology, necessarily entails reflexivity regarding one’s own social location, one’s assumptions about the groups under study, and the tools one is using for measurement and analysis. Conscientiously enacting this level of researcher reflexivity in and of itself marks a departure from mainstream psychology.

In this paper, we engage with these issues while focusing on continental Europe. In many ways, the British Isles are situated between Europe and the U.S., as race is recognized as a social construct, but they share many similar narratives, laws, and norms with the rest of Europe. While we occasionally reference British research, we are choosing to focus nearly exclusively on the continental European context, drawing primarily on examples relevant to ethno-cultural identity development research, as that is the field in which we ourselves are situated. We have divided this paper into four main sections. First, we offer a brief overview of the paradigm of intersectionality and a discussion of existent intersectional identity development work, including potential pitfalls and best practices. Next, we review research and policy worthy of greater attention from developmental scholars interested in conducting intersectional research in continental Europe. We then examine current norms in social category conceptualization and operationalization in continental Europe, offering both critique and points of reflection. In the final section, we delve into both quantitative and qualitative methods, drawing on existing research to offer concrete possibilities for future research.

## Intersectionality

Though the concept of intersectionality can be traced back to the mid-19^th^ century, most prominently to abolitionist and suffragette Sojourner Truth’s 1851 speech “Ain’t I a Woman?,” the word itself was first coined by U.S. legal scholar Crenshaw (1989). In the wake of the Civil Rights Movement and second-wave feminism, Crenshaw and other Black feminist scholar-activists, such as Hooks (1981), Davis (1982), and Collins (1989), laid out legal and experiential arguments pushing academics and practitioners to move beyond single-axis frameworks to account for the differential experiences intersecting inequity creates. This early U.S. work focused primarily on the race-gender nexus, explicating how the interlocking oppressions experienced by Black women can differ both from those of Black men and white women. At the same time, Black feminist scholars in continental Europe were examining similar issues, often in direct conversation with scholars from the U.S., as they critically explored the identities and experiences of Black women in relation to systems of oppression in countries such as the Netherlands (Essed, 1991) and Germany (Oguntoye et al., 1986).

Across continents, the work of intersectional scholars has interrogated normative ontology and epistemology, highlighting the often unquestioned acceptance of knowledge produced by those who are systemically privileged (Essed, 1991; Crenshaw, 1994). Rooted in a social justice orientation, this research problematizes the constructed nature of group boundaries, exposing the processes allowing racialized and gendered oppression to seem natural in everyday notions of what it means to belong to a given group (Collins, 2001). Over the past decades, countless researchers have built on this foundational work, expanding beyond the initial race-gender nexus to examine additional intersecting systems of privilege and oppression along the lines of class, physical ableness, sexual orientation, and religion (e.g., Kessinger, 2008; Taylor et al., 2010; Mirza, 2013; Gökarıksel and Smith, 2017; Walters, 2018; Stojanovski et al., 2019).

Intersectionality was premised on the notion that social constructs have material impact (Crenshaw, 1994; Collins, 2001), including at the individual level. This means that, rather than understanding a social identity category, such as race, as neutral or objective, an intersectional perspective entails an explicit recognition of the power shaping how, when, and in which ways, this construct is applied and to whom. In doing so, intersectionality recognizes the social location a given individual occupies, rather than simply the social categories which may be used to define them (e.g., Collins, 1989). This offers a lens through which to examine the construction of a given social identity category, both in terms of the heterogeneity it may encompass and the fluidity with which it may be applied, enacted, and understood across time and contexts.

### Intersectionality and Identity Development

While we are interested in examining identity development from an intersectional lens, we are conscientiously omitting a discussion of “intersecting identities” in favor of a focus on identities affected by intersecting power, privilege, and oppression (Moradi and Grzanka, 2017). For instance, heterosexual white cisgender men do not innately embody a multiply privileged identity, but instead occupy a social location of systemic, intersecting privilege along the axes of sexual orientation, race, and gender, which is likely to shape their sense of self and broader identity development, and can vary across time and contexts. We intentionally choose this example to underscore the importance of applying the intersectionality framework across populations, including to those regularly situated as unmarked and normative, in opposition to those who are marked and constructed as Other (Yep, 2016). Furthermore, the majority of developmental research across the decades has been conducted by heterosexual white cisgender men, influencing the types of questions asked and masking the power inherent in shaping the research landscape (Syed et al., 2018). Intersectional theory helps to expose essentialized norms of power and privilege, in addition to norms of inequity and oppression, which make it seem as though social categories are indeed innate (Azmitia and Thomas, 2015).

Paralleling work through an intersectional lens, there are additional schools of psychological research which have sought to push against the epistemological and ontological boundaries of positivism, while questioning norms of power and privilege. Critical psychology, which originated in Germany in the 1960s with roots in Marxism, psychoanalysis, and critical theory, took hold in the U.K. in the decades following, where it retains a substantial research base. Critical psychologists explicitly recognize the dynamic interplay between individuals and social structures, and research in this area is historically situated in a social justice perspective (Fine, 2006). The examination of identity through a critical lens in the U.K. has spanned disciplinary boundaries, with epistemological crossover between psychology and fields such as cultural and ethnic studies. British scholars including Hall (e.g., Hall, 1996a,b) and Gilroy (e.g., Gilroy, 1987, 1990) have spearheaded critical scholarship on the construction of race and ethnicity since the 1970s, interrogating essentialist norms and examining identity as performative. This work overlaps with that of British critical psychologists such as Harré and Billig, who led what has been dubbed the discursive turn in psychology in the 1980s (Harré, 2008), focusing on identity construction as individuals position themselves in interaction.

Psychologists continue to build on these critical perspectives, primarily in the social subdiscipline. Among developmentalists, however, there is less of a history of explicitly questioning the construction of social identity categories, as identity is often conceptualized as something located within a given person (for a critical psychology perspective on development, see Morss, 2013). Critically examining identity development, including through an intersectional lens, requires an acknowledgement of identity as socially constructed, fluid, and contextual—and this recognition requires an ontological shift away from positivism. Doing so can include looking “upstream” at macro-level laws, policies, history, and social practices affecting social category membership rather than focusing only on “downstream,” individual-centered explanations (Travis Jr. and Leech, 2014). In doing so, researchers can unearth the constructed nature of social categories themselves, including who they represent and whose experiences are not being represented (Weber and Parra-Medina, 2003; Cole, 2009).

This contextualized focus maps onto recent progress within the broad field of identity development research. For instance, numerous scholars have returned to Erikson’s (1950, 1968) seminal work to advocate for a more nuanced recognition of the dynamic relationship between person and society (Schachter, 2004; Galliher et al., 2017; Rogers, 2018; Syed and Fish, 2018). These scholars are pushing against the mainstream psychological perspective on normative identity development as linear and intrapsychic, instead recognizing its situated, contextual nature. Galliher et al. (2017) explicitly draw on intersectional theory as they put forth a developmental model for contextualized identity research, underscoring the importance of taking into account both the socio-historical *context* as well as the *content* of identity development.

To understand why everyone who identifies as female, for instance, may not engage in identity development processes in the same way requires letting go of a solely intrapersonal operationalization of identity, while also moving away from the positivist goal of generalizability. Both of these aims are in line with the paradigm of intersectionality, and they necessitate an intentional shift in perspective from developmental psychologists (Santos and Toomey, 2018; Syed and Ajayi, 2018). Distinct from other social science disciplines, most psychologists receive little training regarding the epistemological and ontological underpinnings of the research they conduct. In general, researcher objectivity and research generalizability are held in highest esteem as achievable gold standards of practice. That a belief in the *possibility* of objectivity and generalizability reflects a specific research paradigm is rarely discussed in mainstream psychology, and those questioning this gold standard are often relegated to the margins. However, such an interrogation is a necessary first step toward recognizing the power inherent in knowledge construction and the imbalance of what and whose knowledge is considered valid. That said, we do believe it is possible to conduct intersectional, social justice oriented developmental psychology research. One fundamental element of doing so is recognizing the situated nature of both the research itself and the individuals under study—neither development nor developmental science occurs in a vacuum, and our contexts matter.

Galliher et al. (2017) argue that a thorough investigation of content necessarily requires attention to context in order to situate both the micro- and macro-level experiences of participants as they draw meaning from and give meaning to their own identities. From an intersectional perspective, context also necessarily includes the structures of power and privilege shaping everyday lived experience, and, in turn, development. By examining how diverse individuals navigate their identities in relation to their environments and lived experiences, we can also gain a richer understanding of existing process models, which often do not take context into account. To make sure contextualized research does not reiterate existing inequitable social boundaries, however, researchers should attend to the societal norms and expectations that may differentially affect individuals who share certain social categories though not others, taking an intersectional lens to expose heterogeneity within groups.

Moreover, although societal norms and expectations related to privilege and oppression tend to be slow to change (McLean and Syed, 2015), how an individual experiences and understands them is likely to vary as they develop (Azmitia et al., 2008; Hulko, 2009). For example, though children can recognize discriminatory treatment by early adolescence (Brown, 2017), it is only during mid- to late-adolescence that individuals gain the socio-cognitive skills needed to better comprehend discrimination, including being able to situate it socio-historically (Brown and Bigler, 2005; Rivas-Drake et al., 2014). We know that discrimination is linked to a range of deleterious physiological and psychosocial outcomes across populations, including among youth (Huynh and Fuligni, 2010; Benner and Graham, 2013; Schmitt et al., 2014; Benner et al., 2018). Yet, what is less well known is how the development of the socio-cognitive skills needed to comprehend discrimination relates to the skills for understanding one’s own social location within an inequitable world. Do they develop in tandem? Do they develop differentially based on the specific nature of oppression and privilege one experiences? How does context, including family, school, and community, as well as broader policies and histories, fit into this equation? To dig into these questions, we turn to issues salient across Europe today.

## Discrimination and Identity

For children growing up in Europe right now, social cohesion is a key theme in daily life, and questions of identity and belonging are at the core of both popular discourse and empirical research in this realm. Migration within and to the European Union (E.U.) in recent years has added to the existing diversity across the continent, and right-wing, nationalist tendencies have grown in tandem with this trend (Alexander, 2013; Fasel et al., 2013; Erel, 2018). Racism and discrimination are present across societal levels, however, with macro-level policies dynamically reinforcing micro-level interactions (Hatzenbuehler and Link, 2014). As youth navigate their identities, the social context in which they develop matters greatly (Umaña-Taylor et al., 2014). As identity scholars, examining both structural and interpersonal forms of inequity can help us understand the norms, expectations, and boundaries with which youth are grappling as they develop.

A recent representative survey from the 28 European Union member states (O’Flaherty, 2017) found that 38% of respondents, all of whom self-identified as having non-majority heritage, felt discriminated against in their daily lives on the basis of their ethnic or migration background, their skin color, or their religion. Although this survey included sub-sections about specific types of discrimination, it failed to capture the discrimination experienced by those with identities impacted by intersecting systems of oppression. This parallels the majority of psychological research on discrimination, which tends to measure either global or single-axis experiences. Looking at the results from the EU survey (2017), we can see that individuals who identify as Muslim, as women, as being of African heritage, and as second generation experience the highest levels of discrimination. Another recent study found that individuals in Western Europe who identify with multiple minority statuses and have experienced discrimination across multiple domains are more likely than others to perceive discrimination as widespread (Harnois, 2015). Yet, what we cannot see in either of these studies is whether this discrimination is additive, if it differs in nature depending on which social locations one occupies, or how these experiences relate to one’s subjective experience of the prevailing norms and expectations within a given context. To address these questions would require further research from an intersectional lens.

For instance, we know that anti-Muslim discourse in Europe shapes not only everyday interactions but also policies (Yurdakul and Korteweg, 2013; Cowden and Singh, 2017) and norms of belonging (Moffitt et al., 2018). We also know it is deeply intertwined with anti-immigrant sentiment (Reijerse et al., 2013) and that both of these dominant discourses include different norms and expectations based on gender, heritage, and numerous other social categories. Yet, not only do the majority of existing Islamophobia scales (Imhoff and Recker, 2012; Lee et al., 2013) only measure the attitudes of non-Muslims about their perceptions of a Muslim Other, the minority that focus on perceived Islamophobia (Kunst et al., 2013) do not differentiate between the lived realities of Muslim men and women, let alone addressing differential experiences along additional axes of social location.

Although Muslim men in Europe experience regular and systematic discrimination (Barkdull et al., 2011; Holtz et al., 2013; Connor and Koenig, 2015; Giuliani et al., 2018), the myriad laws regulating religious attire affect Muslim women in a way that differs from how they affect either Muslim men or non-Muslim women (Korteweg, 2013; Gohir, 2015). France forbids public school students from primary school through university from wearing any form of religious headscarf, whereas in Germany, many states ban teachers from doing so (Joppke, 2007). In 2010, France was the first European country to ban full face coverings from all public places, with numerous other nations, including Austria and the Netherlands, having since followed suit (Müller, 2019). The stated reasoning behind the various veil bans varies widely, including claims of state neutrality, Christian self-understanding, national security, and gender equality (Byng, 2010; Korteweg, 2013; Helbling, 2014). Yet the laws themselves result in the regulation of the behavior, choices, and life paths of Muslim girls and women, while also normalizing the notion that Muslim women are individuals in need of “protection” through legal regulation (Petzen, 2012).

Thus, these laws have both material and symbolic impact. Drawing on an identity development framework attuned to both individual and society, such as the master narrative framework (McLean and Syed, 2015), developmental scholars could gain important insight into how these laws and the related societal narratives impact identity development across all youth, as they shape norms of belonging and exclusion at the macro- and micro-levels. Similar to recent intersectional work examining how Black youth in the U.S. engage with societal stereotypes as they navigate their identities (Way et al., 2013; Rogers et al., 2015; Way and Rogers, 2015), it would be illuminating to investigate how Muslim girls and women alternately resist, internalize, or otherwise engage with these policies and surrounding narratives. A recent book edited by legal scholar Brems (2014) marked the first major publication to center the voices of women who wear niqab or burka, the face covering veils banned in multiple European nations. The edited collection explores the nuanced decision-making processes and diverse daily experiences of women regularly instrumentalized by politicians and others but whose stories generally remain unheard. Although this book is an extremely important contribution to an under-researched area, it neither investigates identity development nor takes an explicitly intersectional lens.

As far as we are aware, no psychological research has examined how such laws and related societal narratives are perceived by Muslim youth (including girls who do and do not cover their hair) as they develop and navigate their identities. Scholars from other disciplines have argued that not only do headscarf bans create barriers to education and employment for Muslim women (Howard, 2012; Gohir, 2015; Weichselbaumer, 2016), they also reify a fictionally homogeneous Muslim outgroup, underscoring discursive boundaries between “us” and “them” (Yılmaz, 2014). Such normative boundaries undoubtedly play a role in how diverse European youth develop their identities across domains, including in the crucial context of school. Within the ample research into so-called achievement gaps between minority and majority children (e.g., Marx and Stanat, 2012), we are unaware of any research taking a contextual, intersectional lens, which includes policies and narratives directly targeting Muslim women and girls. Intersectional research would help push against the notion of static social identity categories and their use as explanatory variables in deficit oriented research, instead interrogating the ways in which individuals make meaning about their various social locations in relation to their contexts shaped by power and privilege. Understanding how such meaning develops as both individuals and societies change would offer valuable insight into crucial questions of identity construction.

The lacuna of psychological research in this area exposes many interrelated issues, including the ongoing tendency for deficit-oriented work investigating person-level explanations for educational and vocational disparities, the de-valuation of Muslim voices, and the framing of ethnic and cultural diversity as a problem with which (white, Christian) Europe must grapple. The production of meaningful intersectional work in the European context therefore would require a careful unpacking of these tendencies and a recognition of the systems of power currently supporting them as the norm. Borrowing the key questions posed in Syed et al.’s (2018) recent paper on the invisibility of racial/ethnic minorities in U.S. developmental science, those of us doing developmental scholarship in Europe ought to also ask ourselves: From whose vantage point is research conducted? What types of questions are valued? Who gets left out? By digging into these questions, researchers interested in applying an intersectional lens to their work are also more likely to avoid the pitfall of depoliticizing this critical paradigm. Intersectional scholars from across disciplines (Erel et al., 2010; Bilge, 2014; Rosenthal, 2016) have voiced concern about this trend and from our perspective rightly so. Conducting intersectional work cannot simply mean focusing on multiply marginalized populations without addressing the systems upholding such marginalization.

## Measuring and Operationalizing Social Identity Categories

This brings us back to the question of measurement. As researchers born and raised in the U.S. but working in Europe (for varying lengths of time, from periodic to long-term), we, the three co-authors of this paper, have been confronted with our own socialization regarding the social category of “race.” In line with American Psychological Association guidelines (APA, 2017), we each recognize race as a socially constructed concept historically based on oppression and domination, with ongoing psychosocial and material consequences, including at the individual level. As outlined at the beginning of this paper, this does not align with the mainstream European understanding. Instead, across continental Europe, race is understood as an outdated construct which was officially removed from use following WWII in an effort to avoid future genocides and atrocities based on pseudo-scientific arguments casting one group of people as inherently superior to others (Möschel, 2011; Salem and Thompson, 2016). Because of this legal erasure, recognizing “race” is not only taboo, but doing so is logistically and conceptually fraught. Acknowledging race as a social construct separate from but impacted by its colonialist origins and Fascist conceptualization is a conversation far from mainstream continental European discourse, where instead the dominant perspective is one of colorblindness (Lentin, 2008).

Yet, continental Europe is diverse, and racism is deeply embedded at every level of society (O’Flaherty, 2017). So how can diversity, discrimination, and identity be studied if race remains unnamed as a social construct, yet its material and social impact is clear? With expanded citizenship laws following decades of labor and postcolonial migration, new terminology—“migration background”—was adapted in numerous continental European countries to track increasingly diverse polity, based on one’s own place of birth and that of one’s parents and grandparents (Will, 2019). Today, in both popular and academic contexts, the terms “migrant” and “migration background” are often used interchangeably to denote anyone who has migrated, anyone who is the child of immigrants, or, quite often, anyone who is *perceived* as having foreign heritage (El-Tayeb, 2014; Cretton, 2018; Schneider, 2018; Moffitt and Juang, 2019). In both interpersonal contexts and public discourse, such perceptions often fall along the lines of religion and race, with individuals read as Muslim and/or not white labeled “migrants.” Islamic studies scholar Spielhaus (2011) calls this process migrantization—creating a static social category “migrant” detached from actual migration.

This highlights the power imbued in social identity category construction—“migration background” is a fully top-down, ascribed label, and its application and conflation with “migrant” works to naturalize boundaries loaded with material and symbolic impact. Yet, individuals cast as “migrants” are agentic in how they navigate this social reality. So how does this top-down boundary-making play into diverse individuals’ constructions of their identities across the lifespan? Answering this question requires a shift in perspective away from social identity categories as neutral and static, instead recognizing the role of power in shaping the societal structures with which we engage as we develop, and it also requires a shift in research questions toward centering lived experience.

Within psychological research in continental Europe, participants are generally categorized as having a so-called migration background if they or at least one of their parents were not born in the country in which they live. This results in the lumping together of first and second generation individuals from across ethno-cultural backgrounds in opposition to youth of later generations and those of no foreign heritage, reifying this category as static and homogenizing a highly diverse group. Importantly, a recent survey in Germany found that a majority of those officially defined as “persons of migration background” neither identify with this label nor see themselves as migrants (Nesterko and Glaesmer, 2019). Yet, researchers often use the comparative language of “Germans” and “migrants,” to refer to their participants, regardless of place of birth, citizenship, or self-identification of the individuals being studied (Moffitt and Juang, 2019). This common practice fails to capture the lived experiences of youth in Europe today, while reinforcing the discursive division of “us” and “them” discussed above.

From an intersectional perspective, this is problematic for many reasons. For instance, while a child raised in Germany by white, Christian or non-religious immigrant parents may experience bi-cultural identity development in a similar way to other children of immigrants, they will not experience the same interpersonal and institutional discrimination as a child of immigrants from a majority Muslim nation such as Turkey, or the same racialized discrimination as a child of color whose family has resided in Germany for multiple generations and thereby does not officially have a so-called migration background (SVR-Forschungsbereich, 2018). The two latter children in this example will likely experience both interpersonal and systemic discrimination and Othering, including being cast as “migrants” throughout their lives based on their race and religion. The white child of immigrants will benefit from the racialized norm of whiteness associated with Germanness, experiencing power and privilege not afforded to children constructed as Other. In a typical study using “migration background” as a static categorical variable, neither the ascribed nor self-selected identities of these children would be captured, and the impact of racialized discrimination in relation to religion and immigration would remain unexamined (Schwarzenthal et al., manuscript in preparation). This example highlights the situated nature of identity construction, as well as the problems with trading in one static social identity category (e.g., “race”) for another (e.g., “migration background/migrant”) without examining the power structures inherent in the creation of these top-down categories, or the lived experiences of those navigating what it means to be marked as a racialized and/or migrantized Other (Spielhaus, 2011).

There is emerging evidence of a “migrant” identity among some youth in Europe (Svensson and Syed, 2019), but more research is needed to better understand its developmental implications and to situate it in relation to history, policies, and societal norms. While ethnic-racial identity (ERI) has been found sometimes to buffer and sometimes to exacerbate the negative impact of discrimination among ethnic-racial minority youth in the U.S. (with commitment often buffering and exploration often exacerbating) (for a recent meta-analysis, see Yip et al., 2019), how this process works in Europe is even less clear, since ERI is a much more fraught and under-researched concept in this context. To swap out a so-called migrant identity for ERI without a thorough examination of the power structures implicated in the construction of this social category would be doing a disservice to the individuals being studied. Qualitative research has highlighted how class and educational context can impact ethnic identity among Turkish heritage youth in multiple countries, finding that those in more privileged settings tend to emphasize their hybridity while those in less privileged contexts express greater connections to their heritage identities (Faas, 2009). Such work helps illuminate the intersecting nature of oppression and its impact on youth identity development beyond a focus on static identity categories, though myriad additional questions remain regarding broader socio-historical context.

In continental European countries with longer colonialist pasts, such as the Netherlands and France, the centuries of oppression, domination, and dehumanization inherent in these histories, as well as their impact on present policies and narratives, tend to remain invisible in research on identity development. Although the impact of discrimination on adolescent development has been examined by European psychologists for many years (e.g., Verkuyten and Thijs, 2006; Fleischmann et al., 2011; Frankenberg et al., 2013), the ongoing legacy of colonialism tends to go unmentioned. This is not the case in other fields, as postcolonial theory has been developed and applied in diverse settings across Europe for decades. Essed’s (1991) seminal Dutch text on what she dubbed *everyday racism* advocates an examination of the interlocking and differential effects of historical and contemporary oppression, mirroring the work of intersectional scholars in the U.S. from the same period. Yet this work has had little spillover into psychology. As in the U.S. (Syed et al., 2018), the historical and contemporary lack of diversity of scholars in the field^1^ may have contributed to both theoretically and empirically overlooking such central issues within a broader socio-cultural context for ethnic and cultural minority youth.

Although there are many studies in continental Europe comparing various outcome variables across populations of immigrants and their descendants (e.g., Sam et al., 2008; Dimitrova et al., 2016), heritage is generally measured only as a categorical independent variable (e.g., Turkish vs. Moroccan). Within developmental research focusing on within group differences, numerous factors have been examined in the process of identity development. For instance, in a multi-country study, it was found that second generation Turkish heritage adolescents tend to report strengthened ethnic and lowered national identities over time, and that the level of their mothers’ homesickness for Turkey moderated this relation (Spiegler et al., 2019). In line with the rejection-identification hypothesis (Branscombe et al., 1999), there is evidence that stronger ethnic or heritage identity among “migration background” youth in Europe is associated with adaptive psychosocial outcomes (Erentaitė et al., 2018; Schachner et al., 2018). However, broader developmental implications regarding perceived rejection from the national group, or how this relates to policies and laws privileging individuals (perceived as) without a so-called migration background remain little explored.

More broadly, the discourse of “immigrant integration” continues to loom large over research on diversity and identity in Europe (Anthias, 2013), often referencing not just the first generation but the second and third as well. This framing can reinforce a static “us” vs. “them” understanding of national identity (Bhatia and Ram, 2009), while washing over many of the other socio-historical elements of belonging discussed in this section, including the role of power in relation to material structures such as citizenship, and symbolic power as constructed through discourses of ingroup identity. Whether implicit or explicit, the “immigrant integration” discourse tends to shape the questions, methods, and operationalizations used by researchers from across disciplines. By framing ethnic, cultural, and religious diversity in continental Europe as a relatively new “problem” with which fictively homogeneous European nations must contend (Gogolin, 1997; Silverstein, 2005; Schinkel, 2013; Lewicki, 2017), migration and diversity are abstracted from centuries of colonialism and related systems of oppression. As Santos and Toomey (2018) recently argued, the chronosystem is indelibly important to understand context, and in this case, an incorporation of this pertinent history would help move away from deficit-oriented research. In line with the intersectional perspective, we therefore argue that developmental researchers ought to explicitly push against the “immigrant integration” framing. This would require interrogating our own positionalities and questioning the implications of our research frameworks.

Thus, returning to how we began this section, not only are the labels “migrant” and “migration background” highly racialized and often linked to notions of a Muslim Other (El-Tayeb, 2014), their application in research can mask important intersections of oppression and privilege across history and multiple identity domains. By not attending to these differences, a wealth of information regarding diverse youth remains unexamined and little understood, while the power inherent in creating top-down social identity categories remains unquestioned. We are not advocating the insertion of a U.S. American understanding of race into the European context as a fix to this complex problem. What we are arguing, however, is for recognition of the heterogeneity of experience within the quite recently constructed social category of “migration background,” a greater awareness of the power structures at play in relation to the history, policies, and selective application of this label, and their related implications for the identity development of youth in continental Europe today. Developmental psychology research examining how youth navigate racialization and migrantization as they make sense of their interlocking identities is lacking in continental Europe. We believe such work would help center conversations around the roles of power and privilege in both individual development and in developmental science more broadly, and we thus advocate for more research in this realm.

## Future Directions

Numerous scholars in sociology and citizenship studies have begun to examine the processes and outcomes of various forms of solidarity among individuals in Europe situated within the “migrant” social location (Anthias, 2013; Ataç et al., 2016; Cantat, 2016). This work normalizes migration and diversity, centering the agency and identities of individuals marginalized by oppressive citizenship and immigration systems. In line with broader work on coalition building (e.g., Fish and Syed, 2020), examining solidarity in relation to identity development could help make clearer the links between marginalization and cross-group identification. Such work would also highlight diversity within the “migrant” experience while helping to address the third question guiding this special issue regarding common ground among youth experiencing different identity configurations and oppressions. As we noted above, however, we caution against the tendency to use categorical social identity variables as explanatory in and of themselves, as this works to reify a static notion of *migrantness* in opposition to *Europeanness*.

With the aim of capturing a more contextual understanding of identity, some researchers have begun including indices such as the Migrant Integration Policy Index (MIPEX) in their studies, which rates countries based on the openness of their policies related to diversity and immigration. While the MIPEX may help to situate national policies and trends, it does not capture how these are *experienced* by everyday individuals (see Svensson and Syed, 2019). Moreover, it directly supports the aim of “immigrant integration” rather than equity and solidarity. By examining how youth engage with citizenship and immigration policies, as well as related norms and narratives, researchers can unearth otherwise invisible structural boundaries to national belonging, which may be further shaped by additional social identity categories such as gender, religion, and class.

Although citizenship laws across Europe have been overhauled in the past decades, the narratives of ethnic, civic, and cultural national belonging undergirding them link to much longer histories of nation building (Bloemraad et al., 2008; Brubaker, 2009). Recent psychological research has found that individuals who endorse cultural citizenship orientations, meaning they promote a protection of cultural values and symbols, also tend to have strong anti-immigrant attitudes and tend not to support multiculturalism (Reijerse et al., 2013, 2015). Narratives of cultural protection are much more socially acceptable than explicitly racist sentiments, but the systems they uphold and the interpersonal impact they have can be very similar. Situated largely in the tradition of British critical social psychology, there is a growing investigation into everyday productions of citizenship (Barnes et al., 2004; Condor, 2011; Williams, 2013), particularly in relation to an “earned citizenship” discourse (Andreouli and Dashtipour, 2014). Using primarily discursive methods, researchers have examined how individuals engage with mainstream narratives of who is “deserving” of citizenship and national belonging and who is not, highlighting the dynamic interplay between societal expectations and individual identity negotiation (Andreouli, 2013; Andreouli and Dashtipour, 2014; Antonsich, 2015; Moffitt et al., 2018). This work continues in the critical psychology tradition of crossing disciplinary boundaries, drawing on cultural and ethnic studies to recognize the impact of politics and power in identity construction.

This research, while not developmental, emphasizes how structural norms and constraints can create tension within one’s identity, thereby problematizing inequitable systems. However, the emphasis in psychology on consistency, both from researchers and among individuals under study, can result in the ongoing marginalization of identity complexity (Katsiaficas et al., 2011). This means that identities which are in flux as individuals work to resist societal stereotypes (e.g., Way et al., 2013) may be framed not only as conflicting but as problematic. Recognizing that such conflict may embody a developmental aspect of resistance to marginalizing systems can help researchers move away from static conceptualizations of identity categories. Taking this perspective entails a methodological recognition of identity domains as fluid and open to interpretation from both the researcher and participant, an outlook at odds with positivist psychology (Frost, 2009). Yet, doing so allows for an examination of, “how everyday lives are lived on the fault lines of developmental, political, and contextual change and how young people make sense of the coherence of their many selves” (Katsiaficas et al., 2011, p. 134). By engaging in developmental research on modes of resistance to inequitable structures of power, psychologists can draw on the Black feminist foundation of intersectionality (e.g., Collins, 2017), recognizing the value in destabilizing the existing status quo rather than deriding participants for not fitting into it.

### Quantitative Research Through an Intersectional Lens

Regardless of which methods one is using, interrogating the implications of social categories and questioning the value of adapting to inequitable societal systems is extremely valuable. Before offering examples of qualitative frameworks well equipped for examining identity development from an intersectional lens, we will first discuss possibilities using quantitative methods. Quantitative research is generally situated within a (post) positivist perspective, meaning that it aims to uncover an objective reality and strives to be generalizable across populations (Alvesson and Skoldberg, 2009). This seems at odds with intersectionality, a paradigm rooted in the notion that so-called objectivity works to serve inequitable systems of power privileging some to the detriment of others. Yet, there are ways in which quantitative research can help to highlight how systems of privilege and oppression shape individual lived experiences and impact subjective perceptions of what it means to occupy a given social location. Doing so requires asking intersectional questions (Bowleg, 2008), being cognizant and intentional in gathering contextualized data, and being reflexive in data analysis and interpretation (Cole, 2009).

For instance, simply because a participant reports little interpersonal discrimination does not mean that their identities are not shaped by the societal stratification affecting us all. As Bowleg and Bauer (2016) recently noted, broadening our scope in quantitative studies to include system level variables, such as neighborhood SES, violence levels and incarceration rates, air quality levels, and income inequality indices, can help account for systemic inequity impacting individual level outcomes. Including a system-level variable does not imply intersectional research, however. The ways in which such data are analyzed, interpreted, and discussed also matters. Recognizing the historical and political underpinnings of neighborhood segregation, for instance, and including a discussion of the myriad ways it may differentially shape access and opportunity for individuals across social locations can help situate individual experience within a given context while maintaining a social justice perspective.

In terms of capturing experiences specific to individuals’ multiple social locations, researchers must also be attuned to what precisely a given scale is and is not measuring. If scholars are interested in investigating the gendered experiences of interpersonal Islamophobia, for instance, existing measures do not suffice. As with most measures in the field of psychology, the items on existing Islamophobia scales (Imhoff and Recker, 2012; Kunst et al., 2013) were created with the goal of application across populations, rather than aiming to capture the heterogeneity of experiences among individuals positioned within the social category of Muslim.

Without necessarily interrogating the boundaries of the social category itself, one way of addressing this limitation is to develop new scales focusing on specific intersectional experiences. For example, within the past few years, two scales were validated in the U.S. measuring microaggressions, focusing on the experiences of LGBTQ people of color (Balsam et al., 2011) and gendered microaggressions against Black women (Lewis and Neville, 2015). While still operationalizing microaggressions as they were originally defined (Sue et al., 2007; Wong et al., 2014), these scales were created to capture the experiences of discrimination relevant to individuals whose identities are affected by multiple, specific intersections of oppression. These scales mark a step beyond what can be gleaned from an additive or multiplicative conceptualization of intersectionality using interaction effects of existing single-axis scales. Although the use of interaction effects has been advocated for as a way to assess multiple marginalization (Dubrow, 2008; Else-Quest and Hyde, 2016), it has also been heavily criticized (Syed, 2010) because of what it leaves out. By creating new scales, these scholars are recognizing that the nature of discrimination and related societal norms and expectations are often substantively different for LGBTQ white people vs. LGBTQ people of color, and for Black women vs. white women or other women of color. The creation and application of such scales acknowledges the lived reality of the participants whose experiences they aim to capture, thereby helping to center voices often rendered invisible in developmental science (Syed et al., 2018).

Yet, although intersectional measures such as these acknowledge heterogeneity within the social categories of LGBTQ individuals and women, they do little to push against the normative, constructed nature of the categories themselves. One way this has been addressed quantitatively has been to use social identity categories such as race, gender, and sexual orientation not only as independent variables, but instead as outcomes, examining the relevant antecedents and mechanisms comprising these constructs (Helms et al., 2005; Cole, 2009). This can mean the use of scales capturing processes related to group membership, including instances of ascribed versus self-selected categorization, as well as the meaning derived from such instances. Multiple scholars have also advocated measuring the strength of one’s association to social identity groups rather than static group membership (Sarno et al., 2015; Parra and Hastings, 2018). Within Latinx LGBTQ communities, for instance, it has been argued that strong Latinx identity may relate to greater exposure and engagement with anti-LGBTQ norms, whereas a strong LGBTQ identity may help to buffer the negative impact of facing such discrimination (Parra and Hastings, 2018).

### Qualitative Research Through an Intersectional Lens

This brings us to a further discussion of qualitative methods we believe are well equipped to capture identity development through an intersectional lens. We first discuss narrative research, which encompasses multiple methods and approaches, before offering examples of mapping tools and the Photovoice method, both of which are often used within participatory research, and can also be used in conjunction with a narrative approach. Each of these frameworks and methods allows for contextualized, power cognizant, and content-oriented examinations of identity development, while also granting space and visibility to participant voices.

Narrative methods constitute a family of approaches focused on analyzing stories individuals tell about their lives. These stories can be observed in spontaneous everyday conversation or elicited using carefully crafted prompts (Adler et al., 2017). A key difference between narrative and other qualitative methods is that narratives are memories of specific experiences, along with thoughts and emotions imbued by the authors, rather than solely abstract reflections (e.g., “tell me about a time you were treated unfairly” vs. “what do you think about unfair treatment?”). One way that this feature is advantageous in the current context is that through telling stories of past experience, individuals reveal the messy, interrelated details of their lives. As has been long noted in the literature (Hurtado, 1997; Azmitia et al., 2008), intersectionality is not a concept that most people think about and reflect on consciously, and they even have difficultly doing so when asked. Rather, it is through a description of their experiences in various life domains (family, peers, schooling, and work) when the role of intersectionality can become clear. Thus, narrative is particularly well suited to understand identity from an intersectional lens.

Like narrative research, visual mapping tools can also take many forms. In general, mapping as a method entails asking participants to visually depict their own perceptions, experiences, and identities, often in relation to a given place or situation (Katsiaficas et al., 2011). In a recent paper (Futch and Fine, 2014), the history and application of mapping in psychological research was reviewed, highlighting its use as a critical tool for investigating the interplay between subjective experience and social reality. The authors note that researchers should resist the notion that an identity map is a person, instead using maps as a tool for participants to provide their own explanatory narration of why they drew what they drew and how they interpret it (Futch and Fine, 2014). In this way, participants can expound on their social locations, offering insight into links between and across identity domains while also offering greater clarity into how they make sense of policies, narrative, norms, and expectations in the society around them.

Futch, Fine, and colleagues used visual mapping in the years following the 9/11 attacks in the U.S., (Sirin et al., 2008), investigating the ways in which diverse American adolescents engaged with global and interpersonal trauma, stigma, and discrimination as they navigated their religious, national, gender, and academic identities. The participants were asked to draw identity maps at two time points, which the researchers used, along with in-depth interview and survey data, to reach what social scientists refer to as “thick description” (Mills et al., 2010) of the individuals under study. This research thus offers a prime example of how multiple methods can complement one another, capturing youth identity in context, while allowing participants to actively participant in the research process.

Narrative and mapping can both be elements of participatory action research (PAR), another approach that is well-suited to studying intersectionality. A recent example of PAR made use of Photovoice (Wang and Burris, 1997) to understand the experiences of Black, working-class, and LGBT university students in Cape Town (Kessi, 2018). Attending the university where the study was conducted means experiencing an institution established and deeply rooted in a colonial legacy. Kessi aimed to understand how diversely gendered Black students viewed and dealt with the decolonization of the university, in other words, how they understood and challenged key assumptions, such as what knowledge, curriculum, behaviors, mindsets, and identities are valued, prioritized, and taught. Through the use of photos and written narratives, participants described their everyday interactions while reflecting on how these experiences were located within a university institution and societal context defined by specific power hierarchies based on race, class, and gender. These methods allowed students to speak of their experiences of exclusion and belonging that were not confined to a single identity axis but rather richly contextualized. One important finding is that by engaging in this narrative and participatory action research, the students experienced some clarity as to how they were constructing their multidimensional identities within these spaces.

There are myriad other qualitative methods which may offer insight into youth identity development in the European context and elsewhere. We chose to highlight these three families of methods to emphasize the possibility of greater participant voice and engagement within the research process, which may help researchers to make sense of the how intersecting oppression and privilege impact youth as they navigate their developmental paths. Furthermore, each of these types of methods is attuned to both the societal and individual level, allowing researchers to delve into how participants engage with the structures of power in which they are situated.

## Conclusion

In an effort to address the questions guiding this special issue, we found ourselves confronted with two key issues. First, how can intersectional research best be conducted in a context in which the concept of race was officially removed decades ago, yet racialized oppression, in conjunction with migration, citizenship, religion, and other social locations, remains prevalent? Second, how can developmental psychologists adequately engage the social justice oriented, critical paradigm of intersectionality to conduct individual level identity research? Although we certainly do not claim to have offered definitive answers to these questions, we believe that by drawing on methods including those outlined above and taking heed of the issues raised throughout this paper, important, intersectional research can be conducted on youth identity development in contemporary Europe. Moreover, we believe that the time is ripe for such research. As anti-Muslim, anti-immigrant, and ethnic nationalist narratives and policies once again gain prominence (Essed and Nimako, 2006; Meer and Modood, 2009; Fasel et al., 2013; Reijerse et al., 2013; Elchardus and Spruyt, 2014), it is more crucial than ever to understand how diverse youth in Europe navigate intersecting systems of power, oppression, and privilege as they develop their identities.

Over the past years, there have been numerous academic discussions of whether developmental processes among immigrant and minority youth should be studied using the same frameworks as their “majority” peers, or if entirely separate frameworks are necessary (Sam and Berry, 2010; Erentaitė et al., 2018). We are unsure if this is the right question to be asking. Rather than focusing on downstream, individual level differences, we argue for greater inclusion of upstream, system level factors within research on all youth. Doing so would require shifting away from a deficit-oriented perspective in order to, as Godfrey and Burson (2018, p. 22) put it, “focus on marginalizing systems, not marginalized individuals”. This would necessitate a critical examination of the social categories often employed as static and undifferentiated within identity research in continental Europe, namely those of religion, citizenship, and heritage.

The societal creation and application of social identity categories is dynamically related to how individuals shape their own group belonging, both at the individual and collective levels (Smith, 1992; Dede and Addy, 2015). The top-down creation of the label “migration background” has fundamentally altered how researchers in Europe categorize study participants, while also impacting broader discourse. This act alone exemplifies the inequitable power structures differentially shaping lived experience. As racialized individuals are cast as “migrants” in everyday life (El-Tayeb, 2014), based not only on actual migration experience but also on heritage, religion, class, and the intersections between each of these social locations, more research is needed into how youth develop their identities within this current context. This can include more nuanced, contextualized quantitative research to help capture specific processes of oppression and privilege, as well as more qualitative research centering participant voices and helping to untangle how individuals articulate their own experiences navigating identities impacted by intersecting systems of oppression and privilege. In this vein, we look forward to reading future research on intersectional identity development among youth in contemporary continental Europe.

## Author Contributions

All three authors worked together on the conceptualization and initial outlining of the manuscript. UM then completed a first draft, which LJ and MS added to, edited, and gave feedback on. The three authors each worked in turn on the manuscript until it reach its current form.

### Conflict of Interest

The authors declare that the research was conducted in the absence of any commercial or financial relationships that could be construed as a potential conflict of interest.

## Footnotes

^1^This claim is difficult to back up empirically, as race and ethnicity data are not collected in continental Europe, but a recent report from the U.K. (Advance HE, 2018) found that only 0.6% of professors were Black, though Black individuals make up roughly 3.0% of the total population.
